# Multicenter Study of Carbapenemase-Producing *Enterobacterales* in Havana, Cuba, 2016–2021

**DOI:** 10.3390/antibiotics11040514

**Published:** 2022-04-12

**Authors:** Haiyang Yu, María Karla González Molina, Yenisel Carmona Cartaya, Marcia Hart Casares, Meiji Soe Aung, Nobumichi Kobayashi, Dianelys Quiñones Pérez

**Affiliations:** 1 Healthcare-Associated Infections National Laboratory, Pedro Kourí Institute of Tropical Medicine, Havana 11400, Cuba; yuheroyoung@hotmail.com (H.Y.); marikarla@ipk.sld.cu (M.K.G.M.); yeniselc@ipk.sld.cu (Y.C.C.); 2 Microbiology Laboratory, Hermanos Ameijeiras Hospital, Havana 10400, Cuba; mlhart63@yahoo.com; 3 Department of Hygiene, Sapporo Medical University, Sapporo 060-8556, Japan; meijisoeaung@sapmed.ac.jp

**Keywords:** carbapenemase, *Enterobacterales*, Cuba, NDM, *Klebsiella pneumoniae*, *Enterobacter* spp.

## Abstract

Surveillance of carbapenem resistance is particularly important for *Enterobacterales,* mainly in countries with limited healthcare resources. We conducted a cross-sectional study to detect carbapenem-resistant *Enterobacterales* at 10 sentinel hospitals in Havana, Cuba for a six year-period (2016–2021) by the National Reference Laboratory for Health Care-Associated Infections in the Pedro Kourí Institute. A total of 152 isolates were collected with phenotypic production of metallo-β-lactamase. NDM-type carbapenemase was detected in all the 152 isolates, and KPC-type enzyme gene was simultaneously identified in four NDM-positive isolates. The most abundant carbapenemase-producing *Enterobacterales* (CPE) species was *Klebsiella pneumoniae* (69.7%), followed by *Enterobacter cloacae* complex (13.2%), and *Escherichia coli* (5.9%). Over the study period, among CPE, prevalence of *K. pneumoniae* was almost constant, while *Enterobacter* spp. showed slightly increasing tendency. The urinary tract (36.2%) was the most prevalent source of infection with CPE, followed by bloodstream (26.3%) and surgical wound (17.1%), being frequently derived from Intensive Care Units (35.5%) and urology wards (21.7%). This study revealed the present situation of CPE in hospitals in Havana, Cuba, showing the emergence and dissemination of *Enterobacterales* producing NDM-type carbapenemase, mainly *K. pneumoniae*.

## 1. Introduction

In the last two decades, carbapenem-resistant *Enterobacterales* (CRE) infection has become a major public health problem globally, with a high fatality rate and outbreaks [1]. Unfortunately, the COVID-19 pandemic favored the emergence of extremely resistant microorganisms and an increased incidence of resistance to carbapenems, possibly related to the increased use of broad-spectrum antibiotics in patients with COVID-19 [2,3].

In 2017, the WHO placed carbapenem-resistant *Enterobacteriaceae* among the first in its published list of “priority pathogens”, for which epidemiological surveillance and scientific research must be encouraged due to the few or no therapeutic options to treat the infections [4]. Later in the same year, the WHO issued global guidelines for the prevention and control of carbapenem-resistant *Enterobacteriaceae*, *Pseudomonas aeruginosa*, and *Acinetobacter baumannii* complex in healthcare-associated infections in order to reduce their spread and the negative impact of resistance to carbapenems in patients and health care settings [5]. In this context, the implementation of surveillance systems is the first step and a fundamental part of said strategy.

Cuba also has not escaped from the CRE emergency and its clinical, epidemiological, economic and social repercussions. Faced with this call and the growing worldwide multi-drug resistance of the pathogens that cause healthcare-associated infections (HAI), “Pedro Kourí” Institute (IPK) started in 2015 a wide-scale laboratory surveillance of Gram-negative bacilli producing carbapenemases, significant part of which is *Enterobacterales* [6].

The main mechanism causing carbapenems resistance in *Enterobacterales* is the carbapenemase production [7], and according to the Ambler criterion, they are classified into three classes (A, B and D). Class A enzymes (KPC-like) are the most prevalent in the US and Latin American region [8]. On the contrary, class B metallo-β-lactamases (MBLs) (VIM, IMP, NDM) have been increasingly reported in many Latin American countries [9] and class D β-lactamases (OXA), especially the OXA-48, have already been detected in some South American countries [10]. Currently, enzymes from the families *Klebsiella pneumoniae* carbapenemase (KPC), oxacillinase (OXA), New Delhi Metallo-β-lactamase (NDM), Verona Integron Encoded Metallo-β-lactamase (VIM) and Imipenemase (IMP) are those detected most frequently worldwide.

In Cuba, carbapenemase-producing *Enterobacterales* (CPE) was first reported in 2010 in a *K. pneumoniae* clone causing a nosocomial outbreak at Havana [11]. Thereafter, in 2012, the class B enzyme (NDM) was detected in *Acinetobacter soli* [12]. As of 2015, there was a significant increase of CPE, including the geographical spread through the island and the dissemination among different species of gram-negative bacteria [13,14]. Consequently, we decided to start a continuous monitoring which this is the first report. The present study addresses the clinical, microbiological and epidemiological characteristics of CPE causing infections in ten tertiary hospitals in Havana for six years, which includes types of infections, medical treatment sections, bacterial species, antibiograms, as well as the molecular carbapenemase characterization. 

## 2. Results

**Figure 1:**
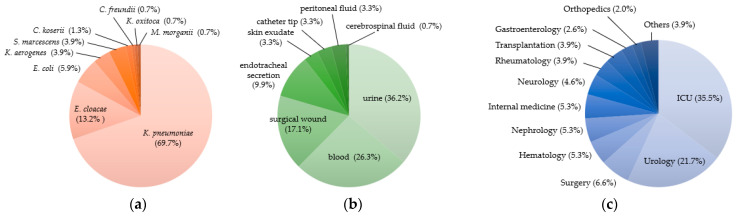
Bacterial species of the 152 CPE isolates (**a**), clinical specimens (**b**) and clinical departments (**c**) from which the CRE isolates were derived (Pedro Kourí Institute, from 2016 to 2021).

**Figure 2:**
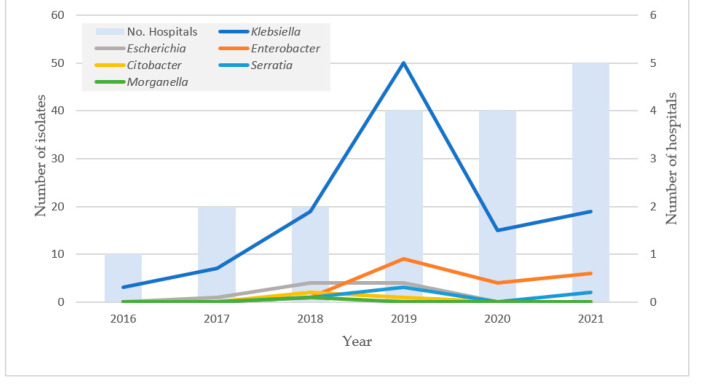
Distribution of CPE genera per year (total 152 isolates) and number of hospitals (total 10 hospitals) (Pedro Kourí Institute, from 2016 to 2021).

During the surveillance period, a total of 357 isolates of *Enterobacterales* were sent to the national reference laboratory in the Pedro Kourí Institute from 10 sentinels’ hospitals in Havana, Cuba. Among them, 152 isolates were phenotypically identified as carbapenemase-producing *Enterobacterales* (CPE) and further characterized. As shown in Figure 1a, *Klebsiella* was the dominant genus of CPE, including the major species *K. pneumoniae* (69.7%), with other minor species (*K. aerogenes* and *K. oxytoca*). Genera of other CPE were *Enterobacter* (*Enterobacter cloacae* complex), *Escherichia* (*E. coli*), *Serratia* (*S. marcescens*), *Citrobacter* (*C. koserii* and *C. freundii*), and *Morganella* (*M. morganii*). During 2016–2021, *Klebsiella* (*K. pneumoniae*, *K. aerogenes*, and *K. oxytoca*) maintained the highest prevalence, with a sharp increase in 2019 (Figure 2). Although prevalence of other genus/species was low, increase in genus *Enterobacter* was noted in the last three years of the study period (2019–2021). Increasing trend was evident for the number of hospitals where CPE was isolated with the passing of years. 

As specimens for CPE, urine was the most common, followed by blood, surgical wound, all of which accounted for approximately 80% (Figure 1b). More than half of CPE isolates (57%) were derived from intensive care unit (ICU) and urology department, while low number of CPE were obtained in various clinical departments (Figure 1c).

Antimicrobial susceptibility patterns of the CPE isolates are shown in Table 1. All the isolates were resistant to ampicillin-sulbactam (SAM), piperacillin-tazobactam (TZP), ceftazidime (CAZ), and cefotaxime (CTX). High resistance rates (more than 70%) were observed against aztreonam (ATM), ciprofloxacin (CIP), gentamicin (GEN), amikacin (AMK), and trimethoprim-sulfamethoxazole (SXT). To fosfomycin (FOS), *E. coli* was mostly susceptible, while other species showed high resistance rates (50–100%). Overall, 73 and 63% of CPE were susceptible to colistin (CST) and tigecycline (TGC), respectively. Resistance to TGC was detected in 11–20% of *Enterobacterales*, except for *K. aerogenes* (all susceptible or intermediate). 

Phenotypically, all the CRE isolates were found to produce metallo-β-lactamase. By immunochromatography (IC), all the isolates (n=152) were positive for NDM, while other carbapenemases (KPC, VIM, OXA-48) were negative in any isolates. Presence of *bla*_NDM_ was confirmed by PCR for the selected 59 IC-positive isolates (43 *K. pneumoniae*, 5 *Enterobacter cloacae* complex, 5 *E. coli*, 2 *K. aerogenes*, one isolate each of *S. marcescens*, *C. koseri*, *C. freundii*, and *M. morganii*), among which KPC gene was also identified in four isolates (2 *K. pneumoniae*, one each of *E. coli* and *K. aerogenes*). 

## 3. Discussion

CRE have become one of the most challenging pathogens in hospital infection control and are increasing at an alarming rate globally [15]. In most cases, the resistance CRE phenotype is associated with the presence of carbapenemase-type beta-lactamases that favor extreme drug resistance, including pan-drug resistance to antibiotics with few or no therapeutic options [16]. Cuba is no exception to this problem, as is revealed in the present study. 

The most prevalent CPE species was *K. pneumoniae* accounting for about 70%, which was consistent with the internationally alarming situation, i.e., the emergence of carbapenemases in *K. pneumoniae* [15]. From 2006 to 2010, resistance to carbapenems in *K. pneumoniae* was sporadic in some countries, however, from 2010 to 2019, countries in the Latin American region reported a gradual but sustained increase, with a wide heterogeneity, reaching above 60% prevalence in some countries [10]. In contrast to the situation in Latin America and the Caribbean, a European monitoring study of urine culture and intra-abdominal cultures (2015–2017) showed that *E. coli* was the predominant species [17].

The clinical departments implicated in our study were ICU, urology, surgery, hematology, and nephrology. ICU-admitted patients have a higher risk to be colonized by antibiotic multiresistant microorganisms, since the antibiotic burden is greater, and they also undergo various invasive manipulations. This favors the selection of resistant strains and the increase in colonization in these departments [18]. By the specimen types, urinary tract infections and bloodstream infections (bacteremia or sepsis) are considered most common infections related to CPE. These infection types are usually associated with intestinal colonization and retrograde spread of bacteria or contact transmission (person-to-person and environment-to-person) [19,20]. The risk of CPE infection is related to individual factors such as length of hospital stay and exposure to invasive procedures. Previous treatments with various antimicrobials in addition to carbapenems, and especially its duration, are essential risk factors for its acquisition as mentioned above [21].

A high prevalence of NDM enzyme associated with low incidence of KPC co-production contrasts with the epidemiology of these enzymes in Latin America and the Caribbean where the KPC-type carbapenemases were widely disseminated in *Enterobacterales* [10]. In China and Europe, a higher prevalence of KPC over NDM is also reported [22,23]. In addition to the location of NDM-type carbapenemase gene on plasmid, the increase in population flow and medical tourism also play an important role in the spread of this carbapenemase gene globally [24]. NDM-type carbapenemase, which was first identified in India (2008), reached the Americas in 2011 [25] and subsequently reported in most Latin American countries [26]. In Cuba, it was first reported in *Acinetobacter soli* in 2012 [12].

NDM-producing bacteria often harbor multiple other resistance genes, and thus they are capable of being resistant to almost all β-lactams and are not inhibited by beta-lactamase inhibitors except AZT. However, co-expression of other resistance genes, such as genes encoding extended-spectrum β-lactamases (ESBLs), AmpC, or another class of carbapenemases (e.g., KPCs) gives rise to resistance to aztreonam [27]. Combining with ribosomal rRNA methylases (16S-RMTases) additionally confers a high level of resistance to all aminoglycosides [28]. All the above limit the use of antimicrobial drugs currently available as monotherapy. The co-expression of different types of carbapenemases, mainly KPC and NDM, as reported in the present study, seems to be further public health concern, associated with the COVID-19 pandemic. 

In the present study, high rate of FOS-resistance was noted in most species of *Enterobacterales*, except for *E. coli*. FOS is a broad-spectrum antimicrobial with bactericidal effect, and considered a potential treatment option against *Enterobacterales* producing ESBL and/or carbapenemase. A main mechanism of FOS resistance in *Enterobacterales* is the acquisition of FOS-modifying enzyme gene *fosA*, which is commonly located on plasmid, transposon, or within integron [29]. This gene has been revealed to co-localize on plasmid with genes ecoding CTX-M type ESBL, NDM-type carbapenemase, or aminoglycoside modifying enzymes. This implies that the spread of FOS-resistance may be presumably associated with ESBL/carbapenemase genes, as well as the use of antimicrobials other than FOS [29]. Therefore, *fosA*-mediated FOS-resistance should be also carefully monitored along with beta-lactamase genes in *Enterobacterales*.

According to the susceptibility to non-carbapenem antibiotics of CPE in the present study, SAM, TZP, ATM, CIP, GEN, AMK, SXT are not recommended in empirical therapy, unless susceptibility to them is previously demonstrated. Because relatively high susceptibility was shown by CST and TGC, combination antibiotic therapy with these antimicrobials may be recommended. Common two-drug combinations include polymyxin plus carbapenems or TGC or FOS [30,31]; TGC plus carbapenems or aminoglycosides or FOS [31,32]; FOS plus aminoglycoside [32]. Triple drug combinations are mainly polymyxin plus TGC and carbapenems if the MIC of the latter is less than or equal to 8 ug/mL [33,34]. There are new antibiotics approved by the FDA in previous years, such as ceftazidime-avibactam in 2015 and meropenem-vaborbactam in 2017, which are active against KPC-type carbapenemases, but not against metallo-β-lactamases [35]. However, these options are expensive and therefore inaccessible to countries with limited health resources.

Given the change in the geographical distribution of carbapenemases, and the emergence and spread of bacteria that produce more than one of these enzymes, the Pan American Health Organization/World Health Organization (PAHO/WHO) emphasizes the importance of proper microbiological diagnosis and the effective and coordinated implementation of infection prevention and control programs, as well as regulations to optimize the use of antimicrobials. The results of the present study revealed the actual situation of CPE in hospitals in Havana, Cuba, during the recent six-year period, showing the emergence and dissemination of NDM-producing *Enterobacterales*, mainly *K. pneumoniae*. This highlights the importance of continuous epidemiological surveillance of carbapenem resistance and their genetic determinants in *Enterobacterales* in medical care facilities.

## 4. Materials and Methods

### 4.1. Bacterial Isolation and Species Identification

Clinical isolates of *Enterobacterales* were collected in ten sentinel hospitals in Havana, as part of the national surveillance of carbapenemases in LNR-IAAS (the National Reference Laboratory for Health Care-Associated Infections) of the Pedro Kourí Institute during the period from 2016 to 2021. The clinical specimens were cultured on McConkey agar (Biolife, Milan, Italy), followed by incubation at 37 °C (Memmert Incubator, Schwabach, Germany) for 18 to 24 h. When a growth of presumptive pathogenic bacteria was confirmed, bacterial species was identified by the conventional method through biochemical tests according to the Manual of Operations of Diagnostic Procedures (MOPD) of the LNR-IAAS. The Kligler and oxidase tests were performed initially and later the following culture media were used for species identification: Simmons citrate agar (Biolife), Christensen urea agar (Biolife), mobility-indole agar (Biolife), malonate sodium (DIFCO), lysine decarboxylase broth (DIFCO) and ornithine decarboxylase broth (DIFCO).

### 4.2. Determination of Antimicrobial Susceptibility

The Epsilon-test (E-test) method was used on Müeller Hinton agar (Oxoid Ltd., Hampshire, UK) to examine susceptibility to SAM, TZP, CAZ, CTX, cefepime, imipenem, meropenem, AMK, GEN, CIP, and TGC, while the disc diffusion method (Kirby-Bauer) (Liofilchem, Roseto degli Abruzzi, Italy) was used for ATM and FOS. CST was evaluated by the disk elution method. The results were interpreted according to the criteria established by the CLSI, 2021 and EUCAST, 2021 [36,37]. Control strains used were *E. coli* ATCC 25,922 and *E. coli* NCTC 13,846 (*mcr-1* positive).

### 4.3. Phenotypic Detection of Carbapenemases

Carbapenemases were phenotypically detected by the commercial method of combined tablets KPC-MBL Confirm ID Pack (Rosco Diagnóstica, Taastrup, Denmark), according to the manufacturer’s instructions. *K. pneumoniae* ATCC BAA-1705-MHT was used as a control strain producing carbapenemase, and *K. pneumoniae* ATCC BAA-1706-MHT was used as a negative control.

### 4.4. Detection of Genetic Types of Carbapenemases

First, immunochromatography (IC) was employed using the commercial kit RESIST-4.0.K.N (CORIS, BioConcept, Gembloux, Belgium), according to the manufacturer’s instructions. In case of negative result, only a red-purple line is observed over the central reading window, at the position of the control line. When result is positive, in addition to a red-purple band in the control line, a visible red-purple band appears in one of the corresponding positions representing OXA-48, KPC, NDM, or VIM.

PCR was applied to the strains with phenotypic production of carbapenemases to determine the presence of the genes encoding KPC, NDM, IMP, VIM, SPM, GIM, SIM, and OXA-48 enzymes. PCR was performed using the primers and conditions described by Poirel et al. (2011) [38].

## Figures and Tables

**Table 1:** Antimicrobial susceptibility rate (%) of CPE isolates (*n* = 152) from 2016 to 2021, Havana, Cuba.

Antimicrobials ^1^	Susceptibility ^2^	Bacterial Species						
		*K. peumoniae*	*Enterobacter cloacae complex*	*E. coli*	*K. aerogenes*	*S. marcescens*	Others ^3^	Total
		(*n* = 106)	(*n* = 20)	(*n* = 9)	(*n* = 6)	*(n = 6)*	(*n* = 5)	(*n* = 152)
SAM	R	100%	100%	100%	100%	100%	100%	100%
TZP	R	100%	100%	100%	100%	100%	100%	100%
MEM	I	2%	0%	0%	17%	0%	20%	3%
	R	98%	100%	100%	83%	100%	80%	97%
IPM	I	4%	5%	0%	17%	0%	20%	5%
	R	96%	95%	100%	83%	100%	80%	95%
ATM	S	1%	10%	11%	17%	0%	20%	4%
	I	0%	0%	11%	0%	0%	0%	1%
	R	99%	90%	78%	83%	100%	80%	95%
CIP	S	8%	10%	33%	0%	17%	0%	10%
	I	1%	0%	0%	0%	0%	0%	1%
	R	91%	90%	67%	100%	83%	100%	89%
GEN	S	5%	5%	11%	0%	0%	0%	5%
	I	2%	0%	0%	0%	0%	0%	1%
	R	93%	95%	89%	100%	100%	100%	94%
AMK	S	8%	10%	22%	0%	0%	0%	8%
	I	2%	0%	11%	0%	0%	0%	2%
	R	90%	90%	67%	100%	100%	100%	90%
SXT	S	3%	0%	22%	0%	17%	0%	4%
	I	0%	0%	0%	0%	0%	0%	0%
	R	97%	100%	78%	100%	83%	100%	96%
FOS	S	22%	20%	78%	0%	17%	20%	24%
	I	4%	5%	0%	0%	33%	0%	5%
	R	74%	75%	22%	100%	50%	80%	71%
CST ^4^	S	75%	75%	100%	67%	-	100%	73%
	I	0%	0%	0%	0%	-	0%	0%
	R	25%	25%	0%	33%	-	0%	27%
TGC	S	58%	75%	67%	83%	66%	80%	63%
	I	27%	10%	22%	17%	17%	0%	23%
	R	15%	15%	11%	0%	17%	20%	14%

^1^ Abbreviation: SAM, ampicillin-sulbactam (SAM); TZP, piperacillin-tazobactam; MEM, meropenem; IPM, imipenem; ATM, aztreonam; CIP, ciprofloxacin; GEN, gentamicin; AMK, amikacin; SXT, trimethoprim-sulfamethoxazole; FOS, fosfomycin; CST, colistin; TGC, tigecycline. ^2^ S, susceptible; I, intermediate; R, resistant. ^3^ others: *C. koseri*, *C. freundii*, *K. oxytoca*, *M. morganii.* ^4^ Susceptibility rate is not shown for *S. marcaescens* and *M. morganii*, because these species are intrinsically resistant to CST.
